# Adaptability and reproducibility of a memory disruption rTMS protocol in the PharmaCog IMI European project

**DOI:** 10.1038/s41598-018-27502-1

**Published:** 2018-06-19

**Authors:** Pablo Martin-Trias, Laura Lanteaume, Elisabeth Solana, Catherine Cassé-Perrot, Sara Fernández-Cabello, Claudio Babiloni, Nicola Marzano, Carme Junqué, Paolo Maria Rossini, Joëlle Micallef, Romain Truillet, Estelle Charles, Elisabeth Jouve, Régis Bordet, Joan Santamaria, Jorge Jovicich, Simone Rossi, Alvaro Pascual-Leone, Olivier Blin, Jill Richardson, David Bartrés-Faz

**Affiliations:** 1 Medical Psychology Unit, Department of Medicine, Faculty of Medicine and Health Sciences, University of Barcelona, Barcelona, Spain; 2 Department of Clinical Pharmacology CIC-CPCET, AP-HM and Institut de Neurosciences des Systèmes (INS) UMR1106, Aix-Marseille University, Marseille, France; 3 Institut d’Investigacions Biomèdiques August Pi i Sunyer (IDIBAPS), Barcelona, Spain; 4 Department of Physiology and Pharmacology, University of Rome “La Sapienza”, Rome, Italy; 5 Department of Neuroscience, IRCCS San Raffaele Pisana, Rome, Italy; 6 IRCCS SDN, Naples, Italy; 7 Department of Geriatrics, Neuroscience & Orthopedics, Catholic University, Policlinic Gemelli, Rome, Italy; 8 University of Lille, Inserm, CHU Lille, U1171, Degenerative and Vascular Cognitive Disorders, Lille, France; 9 Sleep Unit, Neurology Department, Hospital Clinic, Barcelona, Spain; 10 Center for Mind/Brain Sciences (CIMEC), University of Trento, Trento, Italy; 11 Dipartimento di Scienze Mediche, Chirurgiche e Neuroscienze, Brain Investigation & Neuromodulation Laboratory (Si-BIN Lab), University of Siena, Siena, Italy; 12 Berenson-Allen Center for Noninvasive Brain Stimulation and Division of Cognitive Neurology, Department of Neurology, Beth Israel Deaconess Medical Center, Harvard Medical School, MA, 02215 USA; 13 Institut Guttmann de Neurorehabilitacio, Universitat Autonoma de Barcelona, Barcelona, Spain; 14 Neurosciences Therapeutic Area, GlaxoSmithKline R&D, Stevenage, UK

## Abstract

Transcranial magnetic stimulation (TMS) can interfere with cognitive processes, such as transiently impairing memory. As part of a multi-center European project, we investigated the adaptability and reproducibility of a previously published TMS memory interfering protocol in two centers using EEG or fMRI scenarios. Participants were invited to attend three experimental sessions on different days, with sham repetitive TMS (rTMS) applied on day 1 and real rTMS on days 2 and 3. Sixty-eight healthy young men were included. On each experimental day, volunteers were instructed to remember visual pictures while receiving neuronavigated rTMS trains (20 Hz, 900 ms) during picture encoding at the left dorsolateral prefrontal cortex (L-DLPFC) and the vertex. Mixed ANOVA model analyses were performed. rTMS to the L-DLPFC significantly disrupted recognition memory on experimental day 2. No differences were found between centers or between fMRI and EEG recordings. Subjects with lower baseline memory performances were more susceptible to TMS disruption. No stability of TMS-induced memory interference could be demonstrated on day 3. Our data suggests that adapted cognitive rTMS protocols can be implemented in multi-center studies incorporating standardized experimental procedures. However, our center and modality effects analyses lacked sufficient statistical power, hence highlighting the need to conduct further studies with larger samples. In addition, inter and intra-subject variability in response to TMS might limit its application in crossover or longitudinal studies.

## Introduction

Transcranial Magnetic Stimulation (TMS) is a non-invasive technique allowing painless stimulation of the brain, in which brief pulses of current flowing through a coil of wire generate a time-varying magnetic field pulse. The rate of change of the magnetic field determines the induction of a secondary current in a conducting living tissue such as the cortical surface, and this secondary current may lead to the depolarization of the underlying populations of neurons^1^. Although TMS is primarily used in the study of the corticospinal motor system in neurology and neurophysiology^2^, TMS and repetitive TMS (rTMS) have been widely used for many years in the fields of cognitive neuroscience and neuropsychology^3^. Depending on the experimental conditions, TMS can temporarily enhance cognitive functions^4,5^ or, conversely, transiently interfere with major cognitive domains, thereby helping to obtain causal inferences on the role of the stimulated region in behavior. Moreover, TMS can be coupled with information from functional neuroimaging techniques^6^, further enhancing its application in studies on cognitive neuroscience. Imaging information can be used to guide stimulation (increasing the spatial precision of the brain area to be stimulated) and to investigate the effects induced on cerebral networks in terms of their functional reorganization in response to the magnetic pulses and how this relates to a given behavioral outcome.

In this study, we incorporated the use of rTMS into one of the experimental arms within the European Commission Seventh Framework Programme (FP7/2007–2013, grant n° 115009), the Innovative Medicine Initiative’s (IMI) ‘PharmaCog’ project (http://www.imi.europa.eu/content/pharma-cog), which focuses on the early stages of drug development for Alzheimer’s disease (AD)^7^. A series of ‘cognitive challenge experiments’ (including TMS, but also sleep deprivation) were performed in healthy young human volunteers, which involved transiently disrupting cognitive domains relevant to AD. Once the efficacy of the challenge models was established, the reversibility of the induced dysfunction would be tested by employing distinct pharmacological products. Hence, the overarching idea was that the approach would produce experimental platforms that could be used to test for early indications of the efficacy of newly developed drugs.

The aim of the present study was to test the adaptability and reproducibility of a TMS protocol that has been previously reported^2^ to interfere with memory processes. We tested this protocol in two centers using electroencephalogram (EEG) or functional magnetic resonance imaging (fMRI) during the memory recognition phase (see below). To our knowledge, no studies have been published to date that have tested the replicability of the cognitive effects of a TMS protocol in separate experimental sites. The implementation of non-invasive brain stimulation protocols in large clinical trials requires the development of standardized protocols that can be used in multiple centers^8^. Another relevant, but yet untested, aspect for the potential incorporation of TMS protocols in clinical trials involving longitudinal or cross-over designs is to investigate the stability (i.e., test-retest reproducibility) of the observed findings, which was another aim of the present study.

In this study, we compared the effects of rTMS stimulation on recognition memory between the two centers at Marseille (MRS) and Barcelona (BCN) and the two modalities, EEG and fMRI. This manuscript focuses only on the behavioral findings (i.e., memory interference). Putative changes in brain activity/connectivity underlying the observed effects will be analyzed in separate publications.

## Results

### Effects of TMS on reaction times (RTs) during memory encoding and on visual analog scale (VAS) ratings

Although our main outcome variable was memory performance during the recognition phase of the memory task, we also analyzed the putative effects of rTMS on the accuracy of memory encoding, their respective reaction times (RTs) and subjective perception scales. There were no main effects on the accuracy of encoding. For the whole sample (n = 64), RTs were longer when the left dorsolateral prefrontal cortex (L-DLPFC) was under active stimulation only on experimental day 2. However, when focusing on the “sensitive” subsample that participated on both days 2 and 3, there were no differences in the RTs recorded on day 2 between L-DLPFC and vertex stimulation. Regarding the visual analog scale (VAS), volunteers showed less contentment and more annoyance after stimulation on day 2 (see Supplementary Material).

### Main effects of TMS on memory performance (day 1 vs day 2)

Regarding the impact of TMS on recognition memory performance (n = 68), ANOVA showed a main effect for Condition (Hits %: F_(1,64)_ = 11.95, p = 0.001, ηp^2^ = 0.157). A Condition x Time interaction was also observed for this variable (F_(1,64)_ = 14.85, p < 0.0005, ηp^2^ = 0.188). No significant effects were observed for Time (Hits %: F_(1,64)_ = 2.35, p = 0.131, ηp^2^ = 0.035) or for the factors Center (Hits %: F_(1,64)_ = 0.004, p = 0.947, ηp^2^ < 0.0005) or Modality (Hits %: F_(1,64)_ = 0.29, p = 0.589, ηp^2^ = 0.005). These results reveal that active rTMS to the L-DLPFC interfered with memory performance when compared to the stimulation of the vertex at both experimental sites, regardless of whether fMRI or EEG was used.

**Figure 1:**
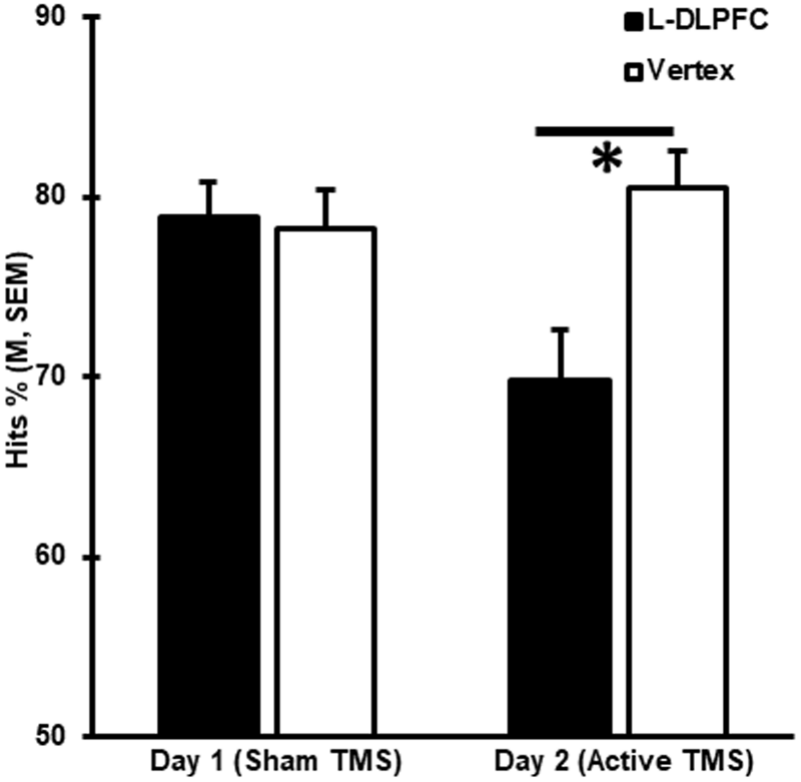
rTMS at the L-DLPFC induced memory interference. Behavioral results from the recognition memory tasks undertaken on experimental days 1 and 2 are shown as mean Hits %. Error bars correspond to the standard error of the mean (SEM).

**Figure 2:**
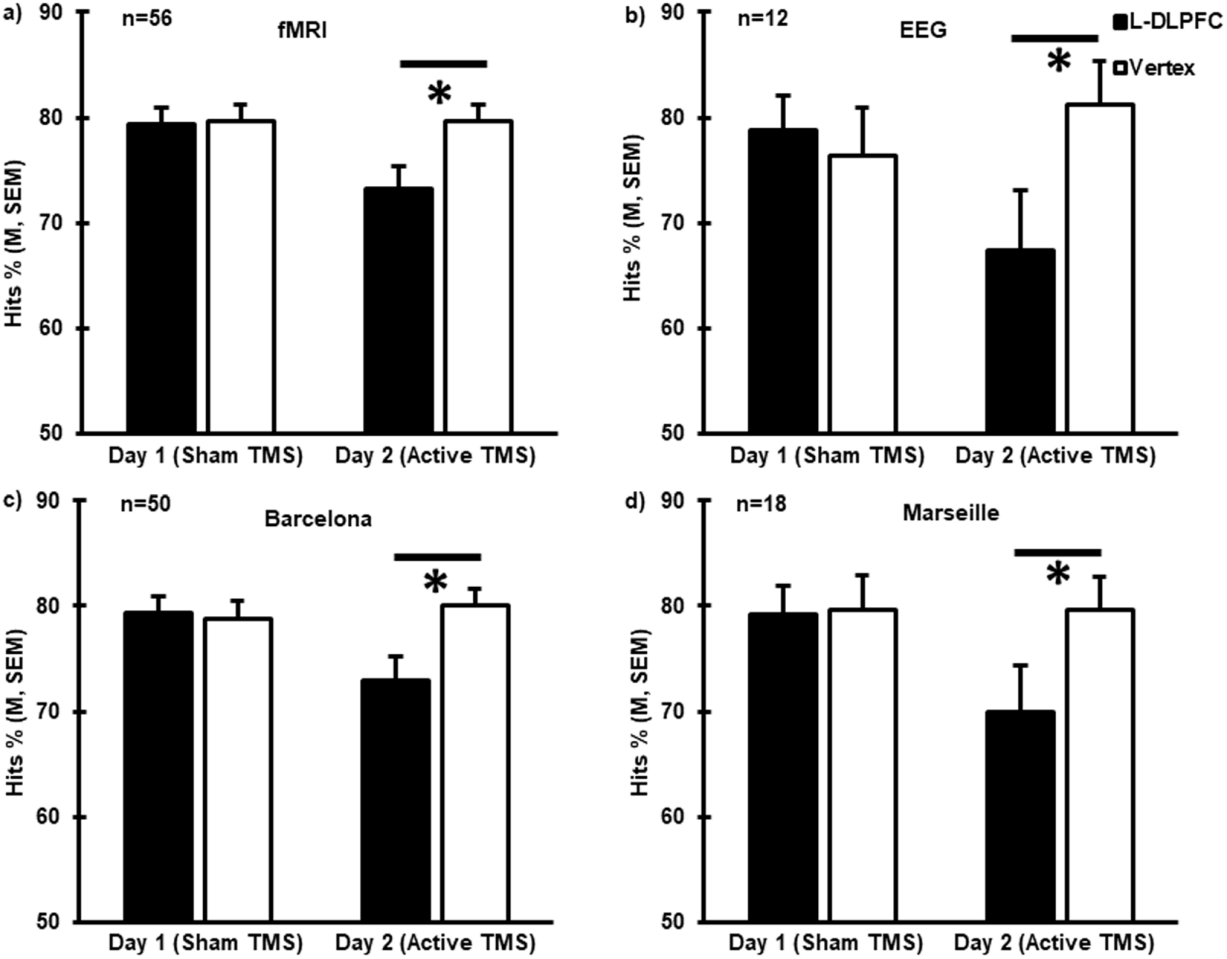
Interference effects of TMS across centers and modalities. Hits % consistently showed a significant reduction in performance in response to rTMS at the L-DLPFC compared to the vertex on day 2 for the: (a) fMRI group (*t_(55)_ = −4.16, p < 0.0005); (b) EEG group (*t_(11)_ = −3.10, p = 0.01); (c) Barcelona group (*t_(49)_ = −3.91, p < 0.0005) and (d) Marseille group (*t_(17)_ = −3.38, p = 0.004). Error bars correspond to SEM.

Post hoc analysis confirmed that memory performance was lower when the L-DLPFC was stimulated than when the vertex was stimulated only on day 2 (Hits %: t_(67)_ = −5.09, p < 0.0005), when active stimulation was applied, but not on day 1 (Hits %: t_(67)_ = 0.16, p < 0.872), when a sham coil was used as placebo (Fig. 1). These trends remained when sub-analyses considering each modality and center were undertaken (Fig. 2).

### Reproducibility of the effects of TMS (day 1 vs day 2 vs day 3)

**Figure 3:**
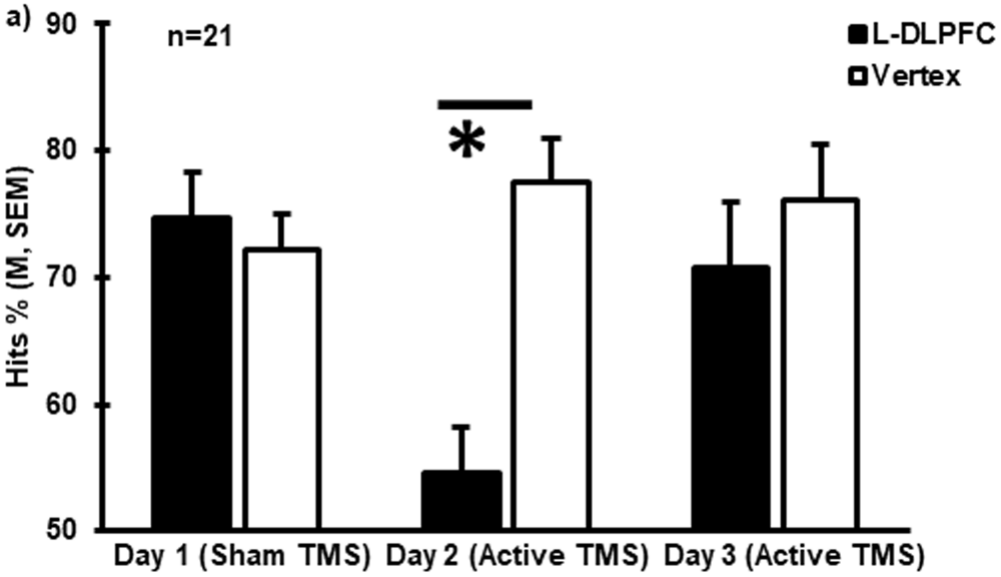
Reproducibility of TMS-induced interference. Behavioral results from the recognition memory tasks undertaken on days 1, 2 and 3 are shown as mean Hits % in a subsample of the subjects (n = 21, p < 0.05). Error bars correspond to SEM.

As described in the Methods, subjects showing the largest response to rTMS interference on day 2 were invited to attend an equivalent session on day 3, which was conducted 15 days later on average. When comparing the data collected from these 21 individuals over the three days, a main effect for Condition was found (Hits %: F_(1,17)_ = 13.09, p = 0.002, ηp^2^ = 0.435). A Time x Condition interaction was also significant (Hits %: F_(1.403,34)_ = 17.05, p < 0.0005, ηp^2^ = 0.501), while there was no main effect for Time (Hits %: F_(2,34)_ = 2.52, p = 0.095, ηp^2^ = 0.129). Post hoc analyses revealed significantly lower memory performance when the L-DLPFC was stimulated than when the vertex was stimulated only for day 2 (Hits %: t_(20)_ = −10.05, p < 0.0005), but not for day 3 (Hits %: t_(20)_ = −0.77, p = 0.448), when active rTMS was also applied, or day 1 (Hits %: t_(20)_ = 0.48, p = 0.634) (Fig. 3). Similar to the main effects observed in the whole sample, we did not observe any significant effects of Center (Hits %: F_(1,17)_ = 1.11, p = 0.306, ηp^2^ = 0.061) or Modality (Hits %: F_(1,17)_ = 0.74, p = 0.402, ηp^2^ = 0.042).

**Figure 4:**
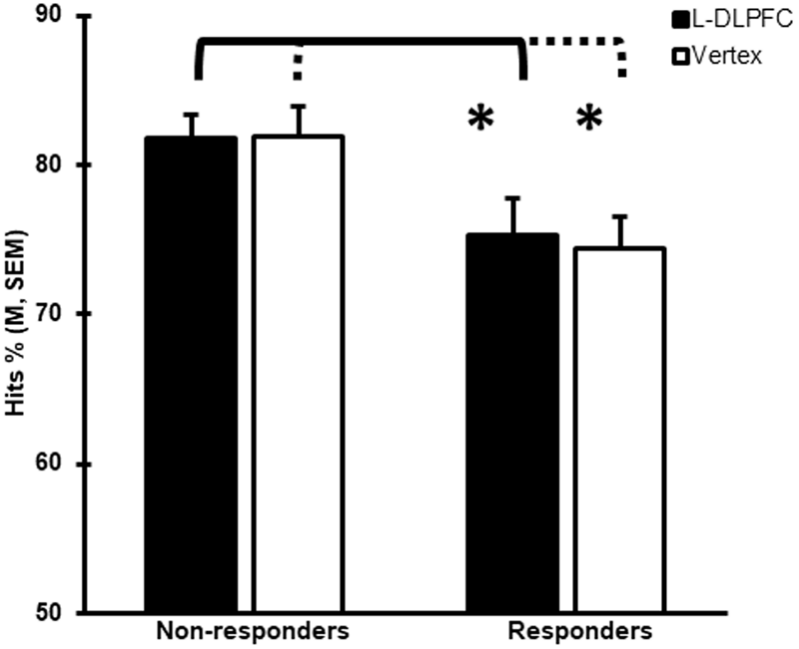
Baseline differences in memory performance (p < 0.05) between non-responders and responders to TMS for both L-DLPFC and vertex stimulation on experimental day 1. Error bars correspond to SEM.

Finally, to identify possible predictors of TMS interference, we compared baseline memory performance (i.e., on day 1 when sham rTMS was used) between the 26 individuals identified as sensitive to TMS (responders) and the 42 subjects who were not sensitive (non-responders) on day 2. We found that the responders exhibited lower memory performance at baseline for both sham conditions (L-DLPFC, Hits %: t_(66)_ = 2.29, p = 0.025; vertex, Hits %: t_(66)_ = 2.53, p = 0.014) than the non-responders (Fig. 4).

Table 1 shows the mean performances and standard deviations (SD) for encoding and recognition for all the dependent variables from experimental days 1, 2 and 3.

**Table 1:** Summary data for all the performance dependent variables. Mean values ± SD for the whole sample included in the ANOVAs performed.

mean ± SD	n	Day 1 (Sham TMS)		Day 2 (Active TMS)		Day 3 (Active TMS)	
		Vertex	L-DLPFC	Vertex	L-DLPFC	Vertex	L-DLPFC
**Encoding**							
Accuracy (%)	65	97 ± 3.5	97 ± 3.6	97 ± 3.6	96 ± 4.7		
	19	96.8 ± 3.7	96.6 ± 4.4	97.4 ± 4.2	94.7 ± 5	97.5 ± 3	97.4 ± 3.8
RT (ms)	64	759 ± 282	751 ± 277	801 ± 247	832 ± 260		
	18	683 ± 187	668 ± 175	708 ± 219	750 ± 246	792 ± 210	844 ± 225
**Recognition**							
Hits (%)	68	79 ± 12.5	79.3 ± 12	79.9 ± 12	72.2 ± 16		
	21	75.2 ± 11	76.4 ± 13	77.6 ± 13	56.7 ± 14	75.4 ± 15	72.5 ± 19
FA (%)	68	10.4 ± 10.1		11.5 ± 10.7			
	21	15.3 ± 11.8		13.4 ± 11		12.8 ± 13	
RT fMRI (ms)	56	1234 ± 311	1249 ± 249	1193 ± 355	1215 ± 366		
	15	1164 ± 191	1191 ± 204	1095 ± 149	1137 ± 180	1128 ± 135	1127 ± 166
RT EEG (ms)	12	724 ± 168	719 ± 209	669 ± 198	716 ± 190		
	5	653 ± 176	602 ± 128	543 ± 111	613 ± 121	621 ± 127	632 ± 138

For reaction times (RTs), data are shown in the Modality subgroups in milliseconds (ms) for the recognition task. RT fMRI corresponds to RTs when fMRI was being acquired, while RT EEG corresponds to RTs under EEG recordings.

## Discussion

In this study, we were able to independently replicate a previously published TMS protocol, although some modifications were made to the original protocol. Thus, overall, our findings suggest that TMS could be incorporated into standardized protocols for multi-center investigations aiming to transiently impair episodic memory in humans. However, we also observed inter-individual variability in response to TMS and failed to demonstrate reproducibility of interference in individuals who had initially responded to TMS.

Over the years, accumulating evidence has unequivocally demonstrated the capacity of non-invasive brain stimulation to modulate cognition in humans^5,9,10^ (for reviews). Despite this, there is a lack of standardized designs and procedures for modulating cognition, which is in sharp contrast to the widely established procedures used to investigate motor cortex functions with TMS^11–13^. Furthermore, in cognitive studies using non-invasive brain stimulation, attempts to replicate findings with published protocols are very scarce. For example, while meta-analytical evidence indicates that a single session of transcranial direct current stimulation (tDCS) modulates linguistic functions^14^, recent attempts to reproduce particular findings have failed to replicate such observations^15^.

To our knowledge, there are very few studies by independent groups that have been explicitly designed to replicate cognitive findings with TMS. To this end, we selected a memory interfering protocol whose effects have been reported in several studies, with overlapping samples used across studies in some cases^16^. Here, we demonstrate that an adaptation of a memory disrupting TMS protocol by two separate research groups was able to confirm the overall expected effects. However it should be noted that the results deriving from the comparison of both center and modality effects, were statistically underpowered in our study. Hence, further studies are needed including larger samples.

In our study, the average reduction in recognition memory induced by L-DLPFC stimulation compared to vertex stimulation was around 7%. This varies from previously reported reductions in performance, which have ranged from 20%^17^ to 24%^18^. The significant effect of TMS detected in this study could be due to the larger sample used here compared to previous studies (i.e., group sizes have typically ranged from 13 to 28 individuals). One of the studies by Rossi *et al*.^16^ used a larger sample (n = 66), but this was divided into two subgroups, old and young subjects. The different magnitudes of the reductions in performance that we observed compared to those of other studies might be also related to the fact that we made some adaptations to the TMS protocol to fulfill PharmaCog project requirements and standards^7^.

**Figure 5:**
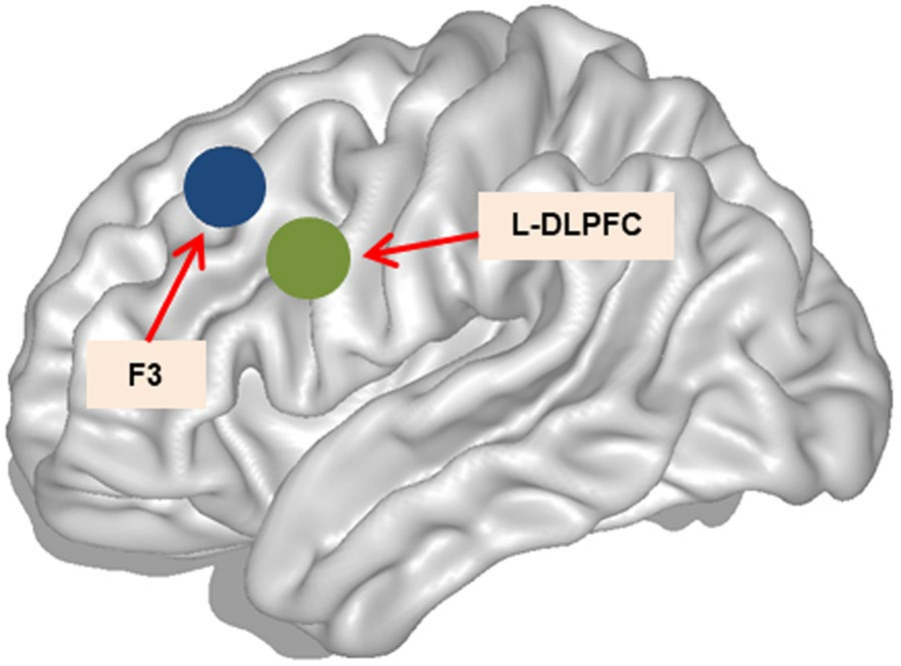
TMS at the L-DLPFC. Left hemisphere sagittal view. Green circle represents the stimulation point for the L-DLPFC, within MNI space coordinates (x, y, z) of −42, 10, 30. Blue circle represents the F3 location, within MNI space coordinates (x, y, z) of −34, 26, 44.

Adaptations of the published protocol included performing TMS using fMRI for guiding instead of anatomical landmark, as earlier studies have indicated that fMRI-guided TMS neuronavigation might produce the strongest behavioral effects (e.g.^19^). Using this approach, the stimulation target was placed posteriorly and laterally within the left middle frontal gyrus (L-MFG) compared to the average estimated F3 standard MNI space^20^ (Fig. 5). Previous studies on memory using TMS have found that the stimulation target might play an important role in the behavioral cognitive outcomes observed. For example, Blumenfeld *et al*.^21^ found that stimulating the left ventrolateral prefrontal cortex (L-VLPFC) before a verbal encoding task produced subsequent memory disruption, whereas TMS to the L-DLPFC facilitated recognition memory compared to vertex stimulation.

Our study sample was composed entirely of men, whereas previous investigations have included both genders. Data from studies using both tDCS and TMS protocols suggest that, overall, females may be more responsive to stimulation than men^22^ for motor^23,24^, visual^25^ and some cognitive domains^26,27^. Accordingly, an unpublished post hoc analysis from the original series in the literature using the adapted protocol found that most of the disruptive effect of TMS was observed in women (*Rossi*, *S*., *personal communication*). Nevertheless, no women were included in the study due to protocol restrictions.

Differences in task difficulty could have also contributed to the smaller reduction in performance that we observed compared to previous studies, as task difficulty is likely to interact with TMS^28,29^. However, overall performance in response to vertex (sham) stimulation was 79.04% (SD = 12.49) of hits in our study, which is comparable to those reported previously (i.e., hits of 74%^17^, 76.2%^30^, 79%^31^ and 72%^18^). Further, it should be noted that subjects were instructed with specific emphasis to intentionally remember the encoded stimulus in our protocol, whereas incidental memory encoding was undertaken in the other studies^16–18^. It has been reported that when considering a semantic level of processing at encoding (e.g., category classification equivalent to indoor/outdoor used in the present model), there are no significant differences in memory or brain activity patterns between intentional and incidental encoding^29,32–35^. However, differences in performance may arise when the level of processing (i.e., deep/semantic *vs* shallow encoding) is specifically manipulated^29,33,36–38^, which was not the case in this study or the previous ones. When comparing overall memory performances between our study and previous reports^17,18^, our results appear to provide further evidence of the importance of ‘level of processing’ over ‘incidental *vs* intentional encoding’ to explain different memory outcomes.

Finally, a direct comparison of the effects of sham and *verum* stimulation of the vertex revealed no differences and no disruption in memory. This was in accordance with previous findings that suggest that the vertex is a valid control for assessing memory function with TMS^17,21,29,39^.

Despite the overall effects observed, only 40% of the participants showed a significant response to TMS interference (i.e., the reduction in memory performance when comparing L-DLPFC to vertex stimulation was at least −1 SD from vertex mean performance). This is consistent with previous results, such as those of López-Alonso *et al*.^40^, who reported responsiveness values reaching 40% in the expected direction depending on the TMS and tDCS protocols used. However, other studies have reported higher rates of responders, including 60% amongst healthy young participants subjected to intermittent theta burst stimulation (iTBS) protocols^41^, 67%^42^, 76%^43^ and 78%^44^ among those subjected to paired-associative stimulation (PAS) protocols, and 75%^45,46^ for those subjected to TMS protocols.

Inter-individual variability is increasingly being recognized as an important factor explaining the findings and discordances in motor studies^11,42^. Inter-subject variability could be due to methodological issues, such as coil orientation^47^, subject characteristics, including age^22,42^ and gender^23,25^, the time of day^48^, genetics^49^, baseline level of excitability^42,50,51^ or short latency intracortical inhibition^40^ (SICI).

As described before, the only variable that predicted TMS response was baseline memory performance. Individuals with lower recognition memory performance at baseline were more susceptible to the disruptive effects of TMS. Indeed, it has been reported that participants with high memory performance at baseline may implement more efficient compensatory processes, making them more resistant to the effects of TMS than those with low memory performance^52^. This observation is consistent with earlier tDCS reports, in which baseline performance was linked to greater positive^53,54^ or negative^55^ cognitive effects of stimulation. It is also in line with a recent transcranial alternating current stimulation (tACS) investigation, in which tACS at the prefrontal cortex increased fluid intelligence capabilities in those with slow baseline performance, but not in those with fast baseline performance^56^. In our study, the larger response to TMS in those with lower baseline memory performance might indicate reduced resilience or less optimal engagement of brain plasticity mechanisms to counteract the effects of TMS. Differences in cognitive reserve, which has been proposed to reflect brain plasticity and is associated with greater efficiency of memory networks^57^, could in principle be associated with the observed differences between the subjects. However, the number of years of education, a common proxy for reserve, and a variable previously shown to interact with the effects of brain stimulation (i.e.^58^), were similar between the responders and non-responders. In any case, determining the mechanisms underlying resilience to the effects of TMS requires neurophysiological data, such as comparisons of brain activity/connectivity patterns during the memory encoding task that was used to guide TMS, which were not available in our study.

We were unable to replicate the initial effects of TMS on memory in the same individuals when they were subjected to TMS again 15 days later. Several studies attempting to reproduce the effects of TMS have reported negative findings^44–46,59–61^, while others have shown stable effects of TMS across different sessions^41,62^. Importantly, none of these reports have assessed reproducibility in the same individuals in distinct experimental sessions.

**Figure 6:**
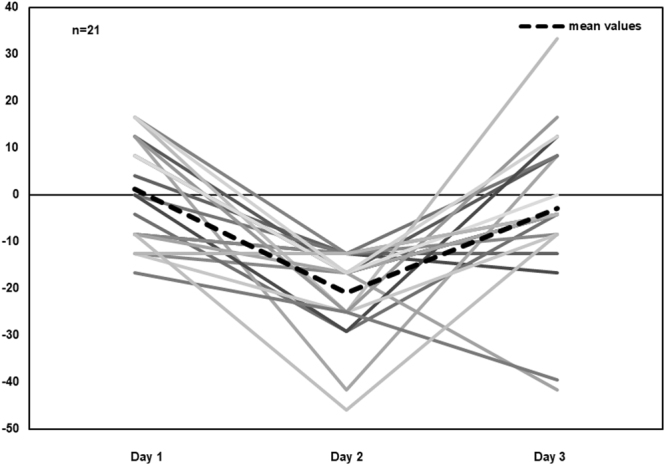
Scatter plot showing individual L-DLPFC-Vertex hits % subtraction (difference) for the subsample that attended all the sessions on days 1, 2 and 3 (n = 21). The thick black dashed line corresponds to the mean values for each day. Negative values correspond to the lower performance in response to L-DLPFC stimulation compared to vertex stimulation.

López-Alonso *et al*.^40^ used different brain stimulation protocols on the same subjects to evaluate intra-subject variability in their responses, reporting that 39%, 45% and 43% of the subjects responded as expected to PAS25, AtDCS and iTBS, respectively, but only 12% of the individuals responded to all the protocols in the expected direction. In our study, we selected 21 individuals who had presented a clear response to TMS for retesting 15 days later, but only 19% (n = 4) of these participants responded consistently to TMS during both experimental sessions. Hence, a particular response at one experimental time point may not be predictive of the same response at a later time point when considering particular individuals (see Fig. 6 for a clear depiction of the performance of the “sensitive” subjects across the experimental sessions). Although our findings indicate a lack of reproducibility of the adapted protocol used, it should be noted that these observations were based only on a smaller subgroup of individuals that attended sessions on both days 2 and 3. Furthermore, the selection of these individuals was biased on purpose as they were specifically selected for their greater response to TMS interference on day 2. This, therefore, does not exclude the possibility that statistical limitations, such as regression to the mean effects, could have been responsible for the lack of effect observed on day 3^63,64^.

Intra-subject variability could have been due to incomplete overlapping of the stimulation site between the sessions^65^, fluctuations in the subjects’ attention within and between sessions^66,67^, the individual’s history of physical activity^68^ or variations in the levels of the stress hormone cortisol^69,70^. In our study, the first possibility seems unlikely to have contributed to our findings as TMS was applied using the same neuronavigated fMRI-based coordinates during the two experimental sessions. By contrast, some attentional biases during rTMS to the DLPFC could have affected our results, as subjective perception of annoyance was increased and contentment decreased after TMS on day 2. Moreover, RTs at encoding were higher when the L-DLPFC was stimulated than when the vertex was stimulated on day 2, which might reflect a distractibility effect on the RTs of responses, but not encoding accuracy. However, it should be noted that this latter effect was not observed when only considering the responders (n = 21). In addition, the blocks of vertex and L-DLPFC stimulation were administered in an interleaving and continuous manner throughout all the experimental sessions. Therefore, as stimulation was not stopped until the end of the encoding task, subjective ratings probably reflected the overall ratings, eclipsing the effects of L-DLPFC and vertex stimulation; thus, the observed changes cannot be specifically attributed to the effect of L-DLPFC stimulation on day 2.

In conclusion, our study replicated an existing cognitive TMS protocol, despite its adaptations for specific experimental purposes. We did not observe any differences in the cognitive effects of TMS between the research center or modality used, but these sub-analyses were underpowered (data not shown). Consequently, further studies with larger samples are needed in order to confirm a lack of center or modality effects. Our data suggest that recruiting individuals exhibiting low baseline memory performance may result in greater observable effects of TMS interference. Stimulating the vertex using either a real or sham TMS coil confirmed that the vertex is a good control for studying visual memory as its stimulation did not disrupt memory. Finally, the effects of TMS could not be reproduced across different time points in a subsample of previously responsive subjects, an issue that needs to be addressed in further TMS investigations.

## Method

### Memory interference task and experimental design

**Figure 7:**
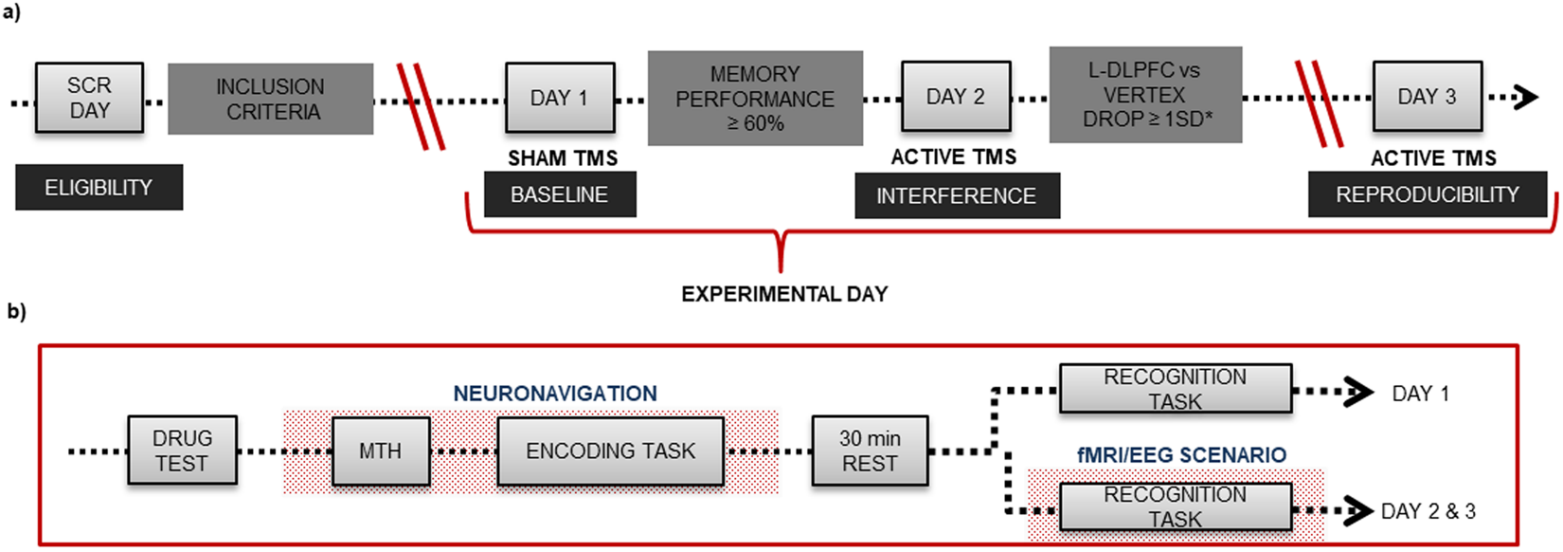
Study design. (**a**) General experimental design with four main time points and requirements that subjects had to meet to complete the whole study. SCR refers to screening. L-DLPFC *vs* VERTEX DROP ≥ 1 SD* refers to a reduction of at least 1 standard deviation (12.5%) in the Recognition performance during L-DLPFC stimulation condition compared to that during vertex stimulation condition. (**b**) Memory task performed on each experimental day (day 1, day 2 and day 3). MTH refers to motor threshold estimation. Drug test consisted of taking a urine sample and a breath test.

Our review of the literature^9^ identified a procedure used in several publications that involved the application of high frequency rTMS during visual memory encoding that disrupted memory performance during a later recognition phase^17^. After some adaptations (see Supplementary Material), we created three equivalent tasks to be undertaken in a counterbalanced order across three experimental days, as depicted in Fig. 7.

On screening day, subjects were familiarized with a short version of the memory task. Subjects had to meet the eligibility criteria based on the inclusion/exclusion criteria detailed in Supplementary Material. During the second visit (experimental day 1), volunteers performed a complete encoding-retrieval memory task while receiving sham TMS. Hence, results from day 1 were used to determine the baseline performance of each individual under sham brain stimulation. Individuals who could not correctly recognize at least 60% of the items (hits % vertex + hits % L-DLPFC/2) were excluded from the final sample. This was done to avoid including individuals who were performing to chance levels, which would correspond to 50% of the performance during recognition memory. On the third day (experimental day 2), selected subjects performed an equivalent version of the encoding-retrieval memory task while subjected to active TMS. Finally, 15 days later, a subsample of subjects exhibiting a decrease in memory performance of at least 1 SD in response to L-DLPFC stimulation when compared to vertex stimulation on day 2 were invited to undergo an identical session (experimental day 3) to test for reproducibility of the effects of TMS. A reduction of −1 SD was considered to reflect a ‘transient memory dysfunction’ induced by TMS. Although a drop of 1.5 SD is used to indicate cognitive impairment in a clinical context, we considered a decrease of 1 SD to be sufficient due to the experimental nature of our study and the fact that it was conducted on healthy young subjects. During both experimental days 2 and 3 (real/active TMS), individuals performed the recognition memory task either inside an MRI scanner or while wearing an EEG cap (see below for the distribution of groups). The study protocol was approved by the French ethics committee “SUD MÉDITERRANÉE I”, the French regulatory authority Agence Nationale de Sécurité du Médicament (ANSM) and the Spanish committee “Comité Ético de Investigación Clínica de l’Hospital Clínic” (CEIC) in Barcelona. The study was in accordance with the Declaration of Helsinki. All volunteers were properly informed and gave written consent. The study was registered in ClinicalTrials.gov for locations in Spain and France (number identifier: NCT01861639, registered on May 23rd 2013).

**Figure 8:**
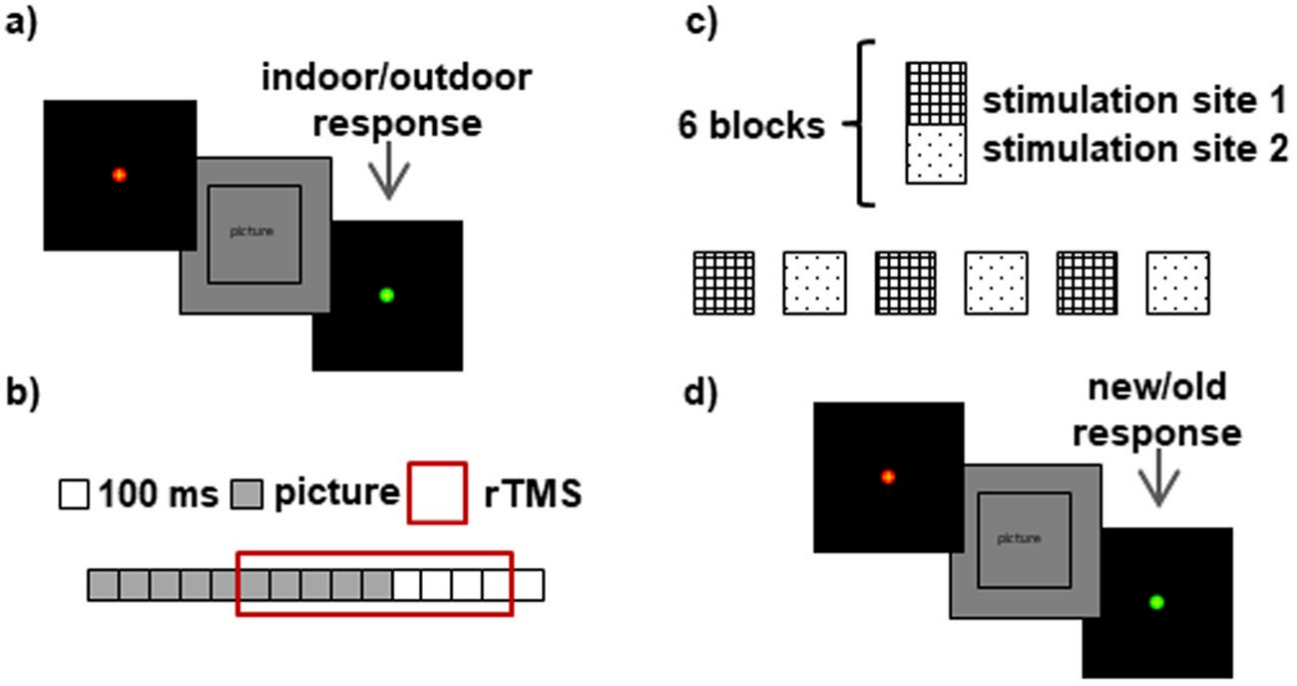
Memory task. In the encoding part, (**a**) each trial consisted of a fixation cross (variable timing), a red cross (warning 1 sec), a picture (1 sec) and a green cross (1 sec). Participants were asked to answer whether the picture was of an indoor or an outdoor scene by pressing the “z” or “m” key, respectively, on a standard computer keyboard after the appearance of the green cross. (**b**) Schematic depiction of the TMS protocol, which consisted of applying a 900-ms rTMS train 500 ms after the appearance of the picture. (**c**) Stimulation was administered in an alternating manner across the 6 blocks of pictures over two brain regions (vertex and L-DLPFC). The order of stimulation was randomly assigned to each subject that remained unchanged across the experimental days. In the recognition part of the task, (**d**) each trial included a fixation cross, a red cross (1 sec), a picture (2 sec) and a green cross (1 sec). Participants were asked to answer if they had seen or not each picture by pressing the “z” or “m” keys on a standard computer keyboard, respectively, or on an MRI-compatible keyboard where the left button corresponded to “saw pictures” and the right button to “did not see pictures”.

The memory encoding task consisted of 6 blocks containing 12 pictures each (50% indoor, 50% outdoor; see Fig. 8a). After a 30-minute break, subjects performed the recognition memory task, in which they were shown 48 new pictures and 24 old pictures during vertex and L-DLPFC stimulation (Fig. 8d). The recognition task was performed in the same experimental room as the encoding task on experimental day 1. On experimental days 2 and 3, subjects performed the recognition memory task in an MRI scanner or while wearing an EEG cap.

### Sample

A total of 68 healthy young individuals (mean age: 24 years; SD: 4) participated in the study, and 21 completed all the three experimental sessions (fMRI group: 56, 44 from BCN; EEG group: 12, 6 from BCN). All the subjects were male due to protocol restrictions (see Supplementary Material).

### MRI-guided TMS protocol

TMS was applied using a MagPro X100 magnetic stimulator (MagVenture A|S, Denmark) combined with an eXimia Navigated Brain Stimulation system (Nexstim, Finland) for the BCN subsample and a Magstim stimulator (Magstim Company Limited, USA, CE certification) combined with the neuronavigation system Brainsight 2.2 (Rogue Research Inc., Montreal, QC, Canada) for the MRS subsample. The resting motor threshold (rMTH) was determined at each experimental session as described in the International Standard Guidelines^71^. High-frequency (20 Hz) 900-ms TMS trains were applied 500 ms after the onset of the picture presentation (this timing of stimulation exerts the clearest effects on memory interference^18^) at a 90% intensity of the individual rMTH. Stimulation was administered in an alternating manner across the 6 blocks of pictures over two brain regions (vertex and L-DLPFC). The order of stimulation was randomly assigned to each subject that remained unchanged across the experimental sessions (see Fig. 8b,c). The vertex site (Cz location according to the 10–20 electrode placement^72^) was used as the control area, while the L-DLPFC site (determined from a previous fMRI memory study briefly described in Supplementary Material; L-DLPFC is widely associated with encoding processes^73,74^) was used as the experimental area. The region corresponds to the intersection between the Brodmann areas 9/46, the boundary between the L-MFG and the left inferior frontal gyrus (L-IFG) mean peak activation voxel according to the Montreal Neurological Institute (MNI) coordinates (x, y, z) of −42, 10, 30 (see Fig. 7). Neuronavigated stimulation with stereotactic registration was performed to ensure accuracy in the localization and position of the TMS coil. To obtain a subjective response to the rTMS administration, we collected responses to visual analog scales (VAS) before and immediately after rTMS administration on experimental days 1, 2 and 3. These VAS scores included ratings for nervousness, contentment, sadness, hope and annoyance. The subjects marked on a 100-mm horizontal line the point that they felt best represented their perception of their current state.

### Data analyses

For encoding, accuracy (defined as the percentage of items correctly categorized as indoor or outdoor) and RTs (defined as the mean reaction times on accuracy responses) were analyzed. Mixed ANOVAs were performed. Different sample sizes were used because of corrupted data (see Table 1). Condition (Vertex *vs* L-DLPFC) and Time (day 1 *vs* day 2) were entered as within-subject factors, while Center (BCN *vs* MRS) was used as a between-subject factor. Modality (EEG *vs* fMRI) was not entered because there were no differences in the protocol for the encoding phase. The same analyses were performed for day 3, but with three Time levels (day 1, day 2 and day 3, n = 19; Bonferroni correction was used for multiple comparisons of the main effects).

For recognition, Hits % (correctly recognized pictures) and RTs (the time elapsed from the presentation of a picture to the subsequent recognition response for each modality subsample) were the main measures of memory performance. Mixed ANOVAs were performed to evaluate the effects of TMS on memory performance (Hits % and RT). Time (two levels: day 1 and day 2) and Condition (two levels: L-DLPFC and Vertex) were entered as within-subject factors, while Center (two levels: MRS and BCN) was considered a between-subject factor. Modality (two levels: EEG and fMRI) was entered as a between-subject factor only for the Hits % ANOVA. For RTs, mixed ANOVA was performed for EEG and fMRI subsamples separately. To test for stability of the effects of TMS, we applied the same statistical model, adding another level to Time (day 1, day 2 and day 3 (n = 21, Bonferroni correction was used for multiple comparisons of the main effects)). The Greenhouse-Geisser correction was used if necessary to correct for non-sphericity. False alarm (FA), which is a false recognized item, was included as a performance index in Table 1, regardless is not a discriminant variable for rTMS effect, but give us a comprehensive perspective of the initial performance level of the included subjects. All effects are reported as significant if p < 0.05. ANOVA’s main effects and interactions were further assessed using post hoc t tests. Data management and analysis were performed using the Statistical Package for the Social Sciences version 17.0 (SPSS Inc.).

To classify subjects as being sensitive to rTMS or not, mean performances during vertex stimulation were considered the benchmark and all performances during L-DLPFC stimulation condition that were below 12.5% of the corresponding performance during vertex stimulation (i.e., corresponding to −1 SD of the group distribution) were considered to be significantly disrupted. Therefore, subjects showing a reduction of at least 1 SD were considered responders and eligible to participate on day 3.

For VAS analysis, we calculated a “change” (after rTMS – before rTMS) for each day and scale, with positive values indicating higher ratings after rTMS. Comparisons for related samples were performed to analyze differences in the subjects’ perceptions between active and sham stimulation.

The datasets generated and/or analyzed in this study are available upon request.

## Electronic supplementary material

Supplementary Information
