# Supplementary material for: Adaptability and reproducibility of a memory disruption rTMS protocol in the PharmaCog IMI European project
Source: Sci Rep. 2018 Jun 19;8:9371. doi: 10.1038/s41598-018-27502-1 (PMC6008461; doi:10.1038/s41598-018-27502-1)
Supplement: Supplementary file 1 — Supplementary Information [file 41598_2018_27502_MOESM1_ESM.docx]

**Adaptability and reproducibility of a memory disruption rTMS protocol in the PharmaCog IMI European project**

Pablo Martin-Trias^1^, Laura Lanteaume^2^, Elisabeth Solana^3^, Catherine Cassé-Perrot^2^, Sara Fernández-Cabello^1^, Claudio Babiloni^4,5^, Nicola Marzano^6^, Carme Junqué^1^, Paolo Maria Rossini^5,7^, Joëlle Micallef^2^, Romain Truillet^2^, Estelle Charles^2^, Elisabeth Jouve^2^, Régis Bordet^8^, Joan Santamaria^3,9^, Jorge Jovicich^10^, Simone Rossi^11^, Alvaro Pascual-Leone^12,13^, Olivier Blin^2^, Jill Richardson^14^, David Bartrés-Faz^1,3,13*^.

**^1^**Medical Psychology Unit, Department of Medicine, Faculty of Medicine and Health Sciences, University of Barcelona, Spain

**^2^** Department of Clinical Pharmacology CIC-CPCET, AP-HM and Institut de Neurosciences des Systèmes (INS) UMR1106, Aix-Marseille University, Marseille, France

**^3^**Institut d'Investigacions Biomèdiques August Pi i Sunyer (IDIBAPS), Barcelona, Spain

**^4^**Department of Physiology and Pharmacology, University of Rome "La Sapienza", Rome, Italy

**^5^**Department of Neuroscience, IRCCS San Raffaele Pisana, Rome, Italy

**^6^**IRCCS SDN, Naples, Italy

**^7^**Department of Geriatrics, Neuroscience & Orthopedics, Catholic University, Policlinic Gemelli, Rome, Italy

**^8^**University of Lille, Inserm, CHU Lille, U1171, Degenerative and Vascular Cognitive Disorders, Lille, France

**^9^**Sleep Unit, Neurology Department, Hospital Clinic, Barcelona, Spain

**^10^**Center for Mind/Brain Sciences (CIMEC), University of Trento, Trento, Italy

**^11^**Dipartimento di Scienze Mediche, Chirurgiche e Neuroscienze, Brain Investigation & Neuromodulation Laboratory (Si-BIN Lab), University of Siena, Siena, Italy

**^12^**Berenson-Allen Center for Noninvasive Brain Stimulation and Division of Cognitive Neurology, Department of Neurology, Beth Israel Deaconess Medical Center, Harvard Medical School, MA 02215, USA

**^13^**Institut Guttmann de Neurorehabilitacio, Universitat Autonoma de Barcelona, Spain

**^14^**Neurosciences Therapeutic Area, GlaxoSmithKline R&D, Stevenage, UK

*dbartres@ub.edu

**Supplementary Material**

**Results:**

*Main effects of TMS on memory encoding performance (day 1* vs *day 2)*

For the memory encoding performance, an analysis of the variance (ANOVA) (n = 65) did not reveal any main effects of TMS on accuracy for Time (F_(1,63)_ = 0.39, p = 0.534, ηp^2^ = 0.006), Condition (F_(1,63)_ = 1.31, p = 0.257, ηp^2^ = 0.020) or the Time X Condition interaction (F_(1,63)_ = 1.63, p = 0.207, ηp^2^ = 0.025). There was also no main effect for Center (F_(1,63)_ = 0.06, p = 0.809, ηp^2^ = 0.001). These findings indicate no general effects of TMS on responses probably corresponding to an attentional component (i.e., identifying by pressing a button if the picture was of an indoor or outdoor scenario) during the memory encoding process.

For the encoding RTs, the same ANOVA as above was performed, but with the exclusion of one subject with outlying values (n = 64). There was a main effect for Time (F_(1,62)_ = 9.34, p = 0.003, ηp^2^ = 0.131) and the Time x Condition interaction (F_(1,62)_ = 6.07, p = 0.017, ηp^2^ = 0.089), but none for Condition (F_(1,62)_ = 3.07, p = 0.085, ηp^2^ = 0.047) nor Center (F_(1,62)_ = 0.52, p = 0.476, ηp^2^ = 0.008). Post hoc analysis (t test for related measures) revealed that significant differences appeared for Condition only on day 2 (t_(65)_ = 2.770, p = 0.007), with RTs being longer for L-DLPFC stimulation than vertex stimulation. There were no differences for Condition on day 1 (t_(64)_ = -0.840, p = 0.404). Thus, this effect seemed to be specific for day 2 (active rTMS), with a longer RT occurring to answer under L-DLPFC stimulation. However, no differences were found for accuracy between L-DLPFC and vertex stimulation.

We also investigated if the observed effects of TMS on recognition memory performance were associated with different reaction times (RT) for each modality. For the EEG subsample (n = 12), there was only an effect for Center (F_(1,10)_ = 12.41, p = 0.006, ηp^2^ = 0.554). Post hoc analysis (t test for independent samples) confirmed that the Marseille subsample had slower RTs than the Barcelona subsample for the day 1 vertex condition (t_(10)_ = 4.892, p = 0.001), the day 1 L-DLPFC condition (t_(8.092)_ = 4.207, p = 0.001) and the day 2 L-DLPFC condition (t_(6.127)_ = 2.500, p = 0.046), but not the day 2 vertex condition (t_(6.010)_ = 2.310, p = 0.06). No differences for Time (F_(1,10)_ = 1.24, p = 0.292, ηp^2^ = 0.110), Condition (F_(1,10)_ = 2.96, p = 0.116, ηp^2^ = 0.228) or the Time x Condition interaction (F_(1,10)_ = 1.98, p = 0.190, ηp^2^ = 0.165) were found. For the fMRI subsample (n = 56), no effects were found for Center (F_(1,54)_ = 0.05, p = 0.829, ηp^2^ = 0.001), Time (F_(1,54)_ = 0.37, p = 0.548, ηp^2^ = 0.007), Condition (F_(1,54)_ = 3.66, p = 0.061, ηp^2^ = 0.063) or the Time x Condition interaction (F_(1,54)_ = 0.13, p = 0.723, ηp^2^ = 0.002).

*Reproducibility of the effects of TMS (day 1* vs *day 2* vs *day 3)*

Among the subsample who attended experimental day 3 (n = 21), we did not observe any main effect on encoding accuracy (data available for n = 19) for Time (F_(2,34)_ = 0.74, p = 0.485, ηp^2^ = 0.042), Condition (F_(1,17)_ = 2.63, p = 0.123, ηp^2^ = 0.134) or the Time x Condition interaction (F_(2,34)_ = 1.58, p = 0.221, ηp^2^ = 0.085). Furthermore, there was no main effect for Center (F_(1,17)_ = 0.25, p = 0.622, ηp^2^ = 0.015).

Regarding the RTs for encoding accuracy (data available for n = 18), a main effect for Time was found (F_(2,32)_ = 5.59, p = 0.008, ηp^2^ = 0.259). Pairwise comparisons revealed significant differences between day 1 *vs* day 3 RTs (p = 0.009), but not for day 1 *vs* Day 2 (p = 0.739) or day 2 *vs* day 3 (p = 0.092) RTs. Post hoc analysis (t test for related samples) showed significant differences in the vertex *vs* L-DLPFC condition RTs for day 3 (t_(18)_ = 2.45, p = 0.025), but not day 1 (t_(18)_ = -0.60, p = 0.553) or day 2 (t_(19)_ = 1.48, p = 0.155). No effects were found for Condition (F_(1,16)_ = 3.22, p = 0.092, ηp^2^ = 0.168), Center (F_(1,16)_ = 0.308, p = 0.587, ηp^2^ = 0.019) or the Time x Condition interaction (F_(2,32)_ = 2.795, p = 0.076, ηp^2^ = 0.149).

For the recognition RTs, the EEG subsample was too small (n = 5) and hence, a mixed ANOVA was performed only for the fMRI subsample (n = 15). There were no main effects for Time (F_(2,26)_ = 2.10, p = 0.143, ηp^2^ = 0.139), Condition (F_(1,13)_ = 4.03, p = 0.066, ηp^2^ = 0.237), Center (F_(1,13)_ = 0.47, p = 0.505, ηp^2^ = 0.035) or the Time x Condition interaction (F_(2,26)_ = 0.56, p = 0.578, ηp^2^ = 0.041).

*Visual analog scale (VAS) analyses (day 1* vs *day 2)*

To determine whether active rTMS induced discomfort compared to sham rTMS, we compared the subjects’ ratings for nervousness, contentment, sadness, hope and annoyance between day 1 and day 2. Since the resulting variables were not normally distributed, a non-parametric test (Wilcoxon signed-rank test) was applied. Significant differences were observed only for contentment (Z = -2.435, p = 0.015), with lower scores obtained on day 2, and annoyance (Z = -2.361, p = 0.018), with higher scores observed on day 2. Thus, our volunteers were less happy and experienced greater discomfort after active stimulation on day 2 compared to sham stimulation on day 1.

*Included vs non-included subject’s analyses*

Finally, non-included subjects after day 1 (n=21) were compared to included participants (N=68) as regards correctly recognized items % (hits % vertex + hits % L-DLPFC/2) and FA %. For Hits comparison, as expected, included individuals exhibited higher performance on day 1 (mean (SD) included subjects: 79.17% (10.31); mean, (SD) non-included: 47.72% (9.4); t-test for independent samples= t_(87)_=12.45, p<0.0005). In contrast, these groups did not differ in FA% variable (mean (SD) included subjects: 10.42% (10.13); mean, (SD) non-included: 6.84% (7.71); t-test for independent samples= t_(87)_=1.49, p=0.141). Therefore, these results suggest that selected subjects showed better memory discrimination rates (better hits performance and comparable FA) than non-selected.

**Methods**

*Original rTMS protocol and its adaptations*

Briefly, the original procedure^1^ consists of presenting emotionally neutral visual pictures during the encoding process. At this stage, individuals have to indicate through pressing a button if the presented stimuli correspond to indoor or outdoor scenes. At the recognition phase, which is tested 20-30 minutes later, some of the previously shown images as well as newly generated pictures are presented and individuals have to provide responses to indicate if they remember (old) or not (new) each picture. Using short trains of high-frequency repetitive TMS (rTMS), previous studies have demonstrated that amongst young individuals, left dorsolateral prefrontal (L-DLPFC) stimulation at the F3 location of the EEG 10-20 system during encoding, reduces the percentage of correctly recognized items by around 20% when compared to sham stimulation, whereas stimulation of the right DLPFC (at the F4 location) induces memory impairment only during the recognition phase^1–4^.

We adapted this original procedure to the experimental needs of the present study. First, the type of memory encoding was implicit or *incidental* in the original studies*,* in that individuals were not explicitly instructed to remember the encoded information for an eventual recognition memory trial. As we administered the task to all the participants on three different occasions (see Fig. 2 in the main text), we were obliged to reveal the explicit nature of the memory task on the first screening day to avoid different encoding strategies between the experimental sessions. In the recognition task for the fMRI subsample, participants were instructed to press the button after the appearance of the green cross, while in the task for the EEG subsample, subjects were instructed to answer after the disappearance of the green cross. Furthermore, our repeated measures design required the creation of three equivalent forms of the task, which needed a greater number of new indoor and outdoor pictures to be generated compared to the original studies. Thus, 744 new neutral pictures with similar characteristics (according to the assessments of 12 raters) were generated and selected, controlling for brightness, quality and size. As described in the literature^5^, we used neuronavigated stimulation to improve the accuracy and replicability of the stimulation point, which was extracted from a previous fMRI study. To target the L-DLPFC area, we used fMRI data results obtained from an independent sample of 12 healthy young male volunteers. Subjects performed an analogous encoding and retrieval fMRI memory task at two separate sessions. A cortical region within the left prefrontal cortex was identified showing robust brain activity at the two separate fMRI sessions during the encoding phase. The peak voxel of maximum activity was used as the stimulation point in the present protocol^6^. Using the structural MRI data of each volunteer acquired on screening day, the reported coordinates were transformed from standard to native space to assign individualized L-DLPFC stimulation points.

*Inclusion/exclusion criteria*

All subjects were right handed (Edinburgh Handedness Inventory >40) and free from major medical conditions according to the Structured Clinical Interview for DSM-IV Disorders and the Mini-International Neuropsychiatric Interview. Normal electroencephalogram (EEG) and structural MRI measurements were required for study participation. Participants were also requested to avoid any drug or medication listed in the TMS safety guidelines^7^. Furthermore, urine samples and a breath test were collected on each experimental day to test for the intake of alcohol or any other drug.

All subjects were male. This, which we recognize limits the generalization of our results, was chosen because an eventual objective of our research project was to compare our TMS findings with those obtained when sleep deprivation was used as a second ‘cognitive challenge’. Gender effects have been described to interact with sleep restriction in their impact on cognitive performance^8^. Moreover, pregnancy is a contraindication to the application of TMS and estrogen fluctuations during the menstrual cycle have been reported to affect working memory fMRI activation^9,10^.

Regarding excluded individuals who could not correctly recognize at least 60% (n=21) we report mean values for correctly recognized items % (hits % vertex + hits % L-DLPFC/2) and FA % to compare them with included subjects (n=68). T-test for independent samples was performed.

**References:**

1. Rossi, S. *et al.* Prefontal cortex in long-term memory : an ‘ interference ’ approach. **4,** (2001).

2. Rossi, S. *et al.* Temporal dynamics of memory trace formation in the human prefrontal cortex. *Cereb. Cortex* **21,** 368–373 (2011).

3. Rossi, S. *et al.* Age-Related Functional Changes of Prefrontal Cortex in Long-Term Memory: A Repetitive Transcranial Magnetic Stimulation Study. *J. Neurosci.* **24,** 7939–7944 (2004).

4. Rossi, S. *et al.* Prefrontal and parietal cortex in human episodic memory: An interference study by repetitive transcranial magnetic stimulation. *Eur. J. Neurosci.* **23,** 793–800 (2006).

5. Sack, A. T. *et al.* Optimizing Functional Accuracy of TMS in Cognitive Studies: A Comparison of Methods. *J. Cogn. Neurosci.* **21,** 207–221 (2009).

6. Martin-trias, P. *et al.* Study of bold stability in resting - state , encoding and recognition memory comparing two fMRI sessions separated in a 15- days interval. Poster presentation. Human Brain Mapping Conference, Hamburg (2014).

7. Rossi, S., Hallett, M., Rossini, P. M., Pascual-Leone, A. & Safety of TMS Consensus Group. Safety, ethical considerations, and application guidelines for the use of transcranial magnetic stimulation in clinical practice and research. *Clin. Neurophysiol.* **120,** 2008–2039 (2009).

8. Cassé-Perrot, C. *et al.* Neurobehavioral and Cognitive Changes Induced by Sleep Deprivation in Healthy Volunteers. *CNS Neurol. Disord. Drug Targets* **15,** 777–801 (2016).

9. Bell, E. C., Willson, M. C., Wilman, A. H., Dave, S. & Silverstone, P. H. Males and females differ in brain activation during cognitive tasks. *Neuroimage* **30,** 529–538 (2006).

10. Shaywitz, S. E. Effect of Estrogen on Brain Activation Patterns in Postmenopausal Women During Working Memory Tasks. *Jama* **281,** 1197 (1999).
